# Supplementary material for: Fish nursery value of algae habitats in temperate coastal reefs
Source: PeerJ. 2019 May 15;7:e6797. doi: 10.7717/peerj.6797 (PMC6525592; doi:10.7717/peerj.6797)
Supplement: Appendix S1 [file peerj-07-6797-s001.docx]

**Appendix 1.**

**Field sampling protocol for the LINKFISH Project**

1. First step: visual census for juvenile fishes

**Diver 1**

- 1. Juveniles of all species will be recorded except those from families having cryptic habits (blenniids, gobiids, etc.). The size of each observed fish will be visually estimated to the nearest cm

2 m

15 m

2. Second step: Measure of habitat characteristics (macro-scale)

**Diver 1**

- 1. “heterogeneity”, defined as cover of the entire transect by different substrate types embedded in the rocky matrix with photophilic algae (sand, gravel, pebbles, *Posidonia*, homogeneous rock)
  2. “complexity” of the rocky substrate including the following indexes:

| Scale | 1 | 2 | 3 | 4 | 5 |
| --- | --- | --- | --- | --- | --- |
| Rugosity |  |  |  |  |  |
| Slope | 0º | 0-15º | 15-40º | 40-65º | >65º |
| Boulders <50 cm | None | Rare | Medium | High | Very high |
| Refuge size | None | Rare | Medium | High | Very high |
| Number of boulders >50 cm | | Classified in three groups 50-100; 100-200; >200 cm | | | |

Scale index description:

- **Rugosity (visual topography estimate of the substratum):**

1. Homogeneous rock without boulders neither refuges.
2. Without boulders >100 cm. The refuges are very scarce.
3. Almost all the boulders are between 100-200 cm but any >200 cm. There are some refuges but all <30 cm.
4. Large number of boulders of different sizes, most of them between 100-200 cm and some >200 cm. The number of refuges is high but <50 cm.
5. Large number of boulders >200 cm and refuges >50 cm.

- **Slope:**


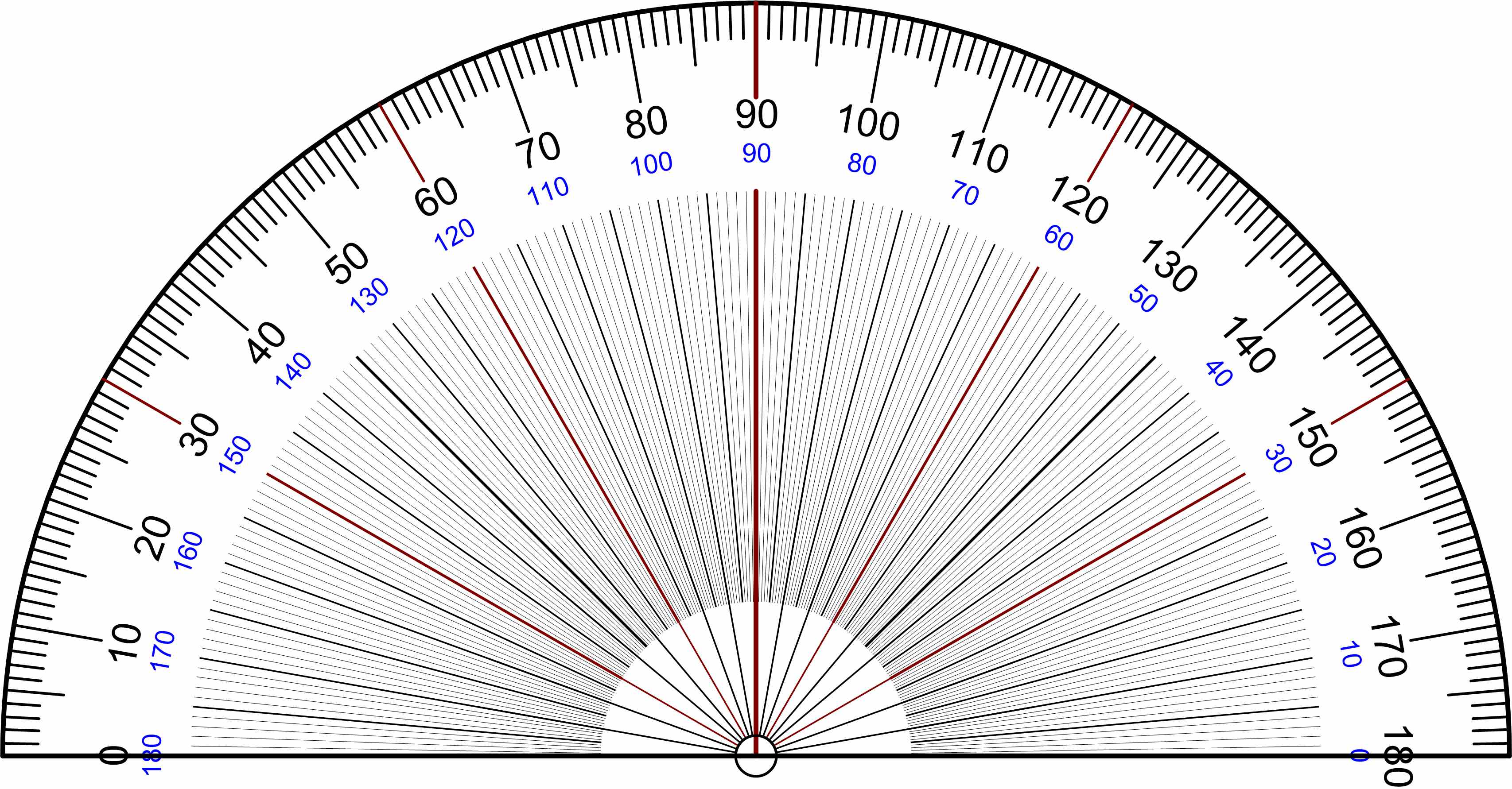


1

2

3

4

5

- **Boulders <50 cm:**

1. Homogeneous rock.
2. Very scarce and concentrated in a small portion of the transect.
3. Common all around the transect homogenously or patchy distributed.
4. Large number of boulders homogenously distributed through the transect.
5. All the transect composed by this type of boulders in high numbers

- **Refuge size (holes in habitat architecture or substratum classified in size categories in cm: <15, 15-30, 30-50, >50)**

1. Homogeneous rock without refuges.
2. Refuges are scarce.
3. Refuges are common patchy distributed.
4. Refuges are very common homogenously distributed through the transect.
5. Large number of refuges homogenously distributed through the transect.

3. Third step: Measure of habitat characteristics (micro-scale)

**Diver 1**

- 1. Algal percent cover in 6 photo-quadrats of 0.4*0.4 cm haphazardly placed on the rocky substrate along the transect to measure the percentage cover of the following algae morphotype (erect tree like, soft leaf like, filamentous, tubular, plumose, bulbus tree like, leathery bands, turf forming) and barens.


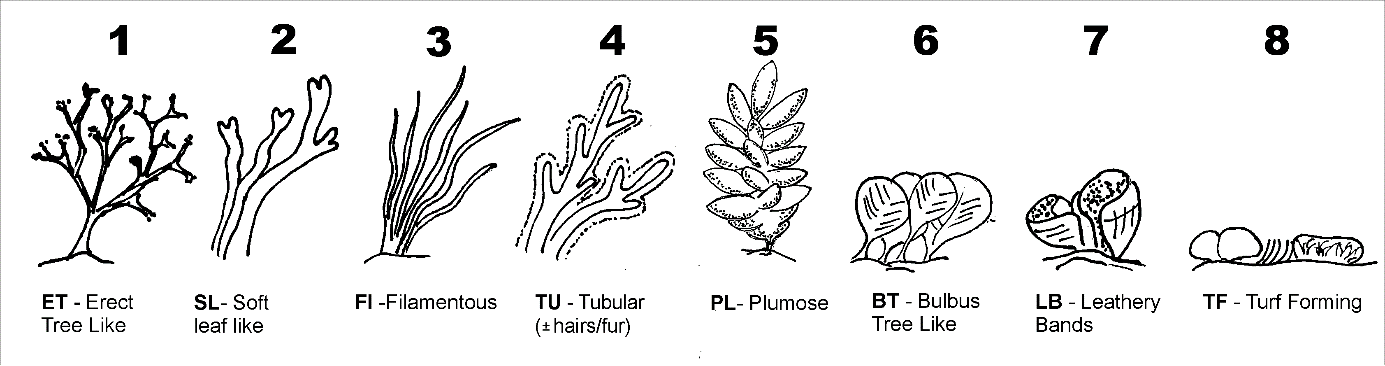


- 1. The transect will be recorded in a video-transect following the tape.

**Diver 2**

- 1. Cover and height of the different algae morphotype defined above in 3 quadrats of 0.5*0.5 cm haphazardly placed on the rocky substrate along the transect.
  2. Number and size of sea urchins and incrusting sponge colonies in 3 quadrats of 1 m^2^ haphazardly placed on the rocky substrate along the transect

4. Four step: Algal biomass and mesograzers

**Diver 1 and 2**

- 1. Algae biomass (wet weight) and mesograzers abundance (by size and functional groups) scraping 3 x 12 cm diameter circular surface area into a fine meshed bag.
