# Supplementary material for: Fish nursery value of algae habitats in temperate coastal reefs
Source: PeerJ. 2019 May 15;7:e6797. doi: 10.7717/peerj.6797 (PMC6525592; doi:10.7717/peerj.6797)
Supplement: Appendix S2 [file peerj-07-6797-s003.docx]

**Appendix 2**. Length weight conversation relationship using power curves (*y=a*x^b^*) of algae associated fauna. Fauna was ranked according to their overall percentage abundance (%Ab.) References used are given in the bibliography below (Açik, 2008; Anderson et al., 1998; Azeiteiro et al., 2001; Baumgärtner and Rothhaupt, 2003; Clark, 1975; Eklöf et al., 2017; Emam et al., 1992; Goodman, 1980; Herman and Heip, 1982; Kneib, 1992; Lasker et al., 1970; Robinson et al., 2010; Rosati et al., 2012; Vassilenko, 1991; Widbom, 1984). The original data and formulas on which the relationships were based on are provided in the associated excel spreadsheet.

| **Species** | **% Ab.** | **a** | **b** | **Adopted or modified from** | **Based on species/Taxa** | **Length measured** |
| --- | --- | --- | --- | --- | --- | --- |
| Harpacticoida | 44.5 | 0.0047 | 2.1780 | Lasker et al. (1970) and Goodman (1980) | Grouped data & *Asellopsis intermedia* | Total length mm |
| Gastropoda | 11.6 | 0.0264 | 2.2950 | Rosati et al. (2012) | *Ecrobia ventrosa* & *Hydrobia* spp. | Total length mm |
| Amphipoda | 10.7 | 0.0056 | 2.4530 | Rosati et al. (2012) & Elkof et al (2016) | *Echinogammarus* spp., *Gammarus insensibilis* & *Gammarus* spp. | Total length mm |
| Bivalvia | 8.3 | 0.0049 | 2.9810 | Rosati et al. (2012) & Elkof et al (2016) | *Dreissena* sp., *Mytilus edulis* & *Limicola balthica* | Total length mm |
| Polychaeta | 6.5 | 0.0125 | 1.6400 | Rosati et al. (2012) | *Hediste diversicolor, Ficopomatus enigmaticus* & Nereididae | Total length mm |
| Ostracoda | 5.0 | 0.0166 | 1.9280 | Anderson et al (1998) & Herman & Heip (1982) | *Physocypria pustulosa*, *Cypridopsis vidua* & *Cyprideis torosa* | Total length mm |
| Caprellidae | 3.3 | 0.0039 | 2.4747 | Vassilenko (1991) | *Caprella cristibrachium, C. kroyeri, C. penantis* & *C. bispinosa* | Total length mm |
| Acari | 2.5 | 0.0829 | 1.7467 | Baumgartner &Rothhaupt (2003) | Hydracarina | Total length mm |
| Nematoda | 1.8 | 0.0056 | 1.1985 | Widbom (1984) | Nematoda | Total length mm |
| Isopoda | 1.3 | 0.0088 | 2.5960 | Rosati et al. (2012) | *Asellus sp., Lekanesphaera hookeri, Lekanesphaeran monodi* | Total length mm |
| Diptera | 1.1 | 0.0010 | 2.6671 | Rosati et al. (2012) | Ceratopogonidae, *Chironomus plumosus, Chironomus sp., Diamesinae, Stratiomyidae, Tabanidae & Tanypodinae* | Total length mm |
| Tanaidacea | 0.9 | 0.0027 | 2.8890 | Kneib et al. (1992) | *Hargeria rapax* | Total length mm |
| Amphiura | 0.8 | 0.1186 | 2.2760 | Robinson et al. (2010) | *Amphiura chiajei* | Disc diameter mm |
| Cumacea | 0.3 | 0.0022 | 2.4200 | Rosati et al. (2012) | Cumacea | Total length mm |
| Natantia | 0.3 | 0.0019 | 3.0330 | Robinson et al. (2010) | *Processa noveli holthusisi* | Total length mm |
| Galathea | 0.2 | 0.0896 | 2.6000 | Robinson et al. (2010) | *Galathe nexa* | Total length mm |
| Pantopoda | 0.2 | 0.0039 | 2.4747 | Non found | Used conversion of Caprellidae | Total length mm |
| Opisthobranchia | 0.1 | 0.0404 | 2.1937 | Clark et al. (1975) | *Alderia modesta, Polycera dubia & Polyerella emertoni* | Total length mm |
| Nudibranchia | 0.1 | 0.0404 | 2.1937 | Clark et al. (1975) | *Alderia modesta, Polycera dubia & Polyerella emertoni* | Total length mm |
| Sipunculidae | 0.1 | 0.0118 | 2.2300 | Acik (2008) | *Golfingia vulgaris, Aspidosiphon misakiensis & Phascolosoma agassizii* | Total length mm |
| Asteroidae | 0.0 | 0.0283 | 2.1930 | Robinson et al. (2010) | *Leptasterias muelleri* | Total length mm (arm tip to opposite arm tip) |
| Paguridae | 0.0 | 0.0155 | 2.6110 | Robinson et al. (2010) | *Pagurus bernhardus* | Total length mm |
| Alpheidae | 0.0 | 0.0319 | 2.1756 | Robinson et al. (2010) | *Calocaris macandreae* | Total length mm |
| Pisa | 0.0 | 0.1157 | 2.9140 | Robinson et al. (2010) | *Eurynome aspersa* | Width carapace |
| Portunidae | 0.0 | 0.0576 | 2.8750 | Robinson et al. (2010) | *Liocarcinus holsatus* | Width carapace |
| Macropodia | 0.0 | 0.2921 | 2.2900 | Robinson et al (2010) | *Macropida rostrata* | Width carapace |
| Echinoidea | 0.0 | 0.0700 | 2.1470 | Robinson et al (2010) | *Echinocyamus pusillus* | Total length |
| Decapoda | 0.0 | 0.0019 | 3.0330 | Robinson et al. (2010) | *Processa noveli holthusisi* | Total length |
| Polyplacophora | 0.0 | 0.1949 | 2.5244 | Emam et al. (1992) | *Acanthopleura spiniger* | Total length |
| Ethusidae | 0.0 | 0.1157 | 2.9140 | Robinson et al. (2010) | *Eurynome aspersa* | Width carapace |
| Majidae | 0.0 | 0.1157 | 2.9140 | Robinson et al. (2010) | *Eurynome aspersa* | Width carapace |
| Mysidacea | 0.0 | 0.0020 | 3.0298 | Azeiteiro et al. (2001) | *Mesopodopsis slabberi* | Total length |
| Platyhelminthes | 0.0 | 0.0404 | 2.1937 | Non found | Used conversion of Nudibranchia | Total length |
| Inachidae | 0.0 | 0.2658 | 2.5410 | Robinson et al. (2010) | *Inachus dosettensis* | Width carapace |
| Holothurioidea | 0.0 | 0.0118 | 2.2300 | Non found | Used conversion of Sipunculids | Total length |

**References conversion:**

Açik, S. (2008). Sipunculans along the Aegean coast of Turkey. *Zootaxa*, 21–36.

Anderson, D. H., Darring, S., and Benke, A. C. (1998). Growth of Crustacean Meiofauna in a Forested Floodplain Swamp: Implications for Biomass Turnover. *J. North Am. Benthol. Soc.* 17, 21–36.

Azeiteiro, U. M., Fonseca, J., and Marques, J. C. S. (2001). Biometry, estimates of production and seasonal variation in the biochemical composition of Mesopodopsis slabberi (Van Beneden, 1861) (Crustacea: Mysidacea). *Bol. Inst. Esp. Ocean.* 17, 15–25.

Baumgärtner, D., and Rothhaupt, K. O. (2003). Predictive Length-Dry Mass Regressions for Freshwater Invertebrates in a Pre-Alpine Lake Littoral. *Int. Rev. Hydrobiol.* 88, 453–463..

Clark, K. B. (1975). Nudibranch life cycles in the Northwest Atlantic and their relationship to the ecology of fouling communities. *Helgoländer Wissenschaftliche Meeresuntersuchungen* 27, 28–69..

Eklöf, J., Austin, Å., Bergström, U., Donadi, S., Eriksson, B. D. H. K., Hansen, J., et al. (2017). Size matters: relationships between body size and body mass of common coastal, aquatic invertebrates in the Baltic Sea. *PeerJ* 5, e2906..

Emam, W. M., Ismail, N. S., and Abou Gabal, M. N. (1992). Age and growth of chiton Acanthoplura spinger from the northwertesn region of the Red Sea. *Indian J. Mar. Sci.* 21, 274–277.

Goodman, K. S. (1980). The estimation of individual dry weight and standing crop of harpacticoid copepods. *Hydrobiologia* 72, 253–259.

Herman, P. M. J., and Heip, C. (1982). Growth and respiration of Cyprideis torosa Jones 1850 (Crustacea Ostracoda). *Oecologia* 54, 300–303.

Kneib, R. T. (1992). Population dynamics of the tanaid Hargeria rapax (Crustacea: Peracarida) in a tidal marsh. *Mar. Biol.* 113, 437–445. doi:10.1007/BF00349169.

Lasker, R., Wells, J. B. J., and McIntyre, A. D. (1970). Growth, reproduction, respiration and carbon utilization of the sand-dwelling harpacticoid copepod, *Asellopsis intermedia*. *J. Mar. Biol. Assoc. UK* 50, 147–160.

Robinson, L. A., Greenstreet, S. P. R., Reiss, H., Callaway, R., Craeymeersch, J., De Boois, I., et al. (2010). Length-weight relationships of 216 North Sea benthic invertebrates and fish. *J. Mar. Biol. Assoc. United Kingdom* 90, 95–104.

Rosati, I., Barbone, E., and Basset, A. (2012). Length-mass relationships for transitional water benthic macroinvertebrates in Mediterranean and Black Sea ecosystems. *Estuar. Coast. Shelf Sci.* 113, 231–239.

Vassilenko, S. V. (1991). Eco-physiological characteristic of some common caprellid species in the Possjet Bay (the Japan Sea). *Hydrobiologia* 223, 181–187.

Widbom, B. (1984). Determination of average individual dry weights and ash-free dry weights in different sieve fractions of marine meiofauna. *Mar. Biol.* 84, 101–108.
