# Supplementary material for: Fish nursery value of algae habitats in temperate coastal reefs
Source: PeerJ. 2019 May 15;7:e6797. doi: 10.7717/peerj.6797 (PMC6525592; doi:10.7717/peerj.6797)
Supplement: Table S1 — PCA eigenvalues and percentage and cumulative percentage of variation explained by PCA axis in algae morphotype cover over dive transects corresponding to Fig. 3. [file peerj-07-6797-s008.docx]

S1 PCA eigenvalues and percentage and cumulative percentage of variation explained by PCA axis in algae morphotype cover over dive transects corresponding to figure 3.

| PC | Eigenvalues | %Variation | Cum.% Variation |
| --- | --- | --- | --- |
| 1 | 37.9 | 34.2 | 34.2 |
| 2 | 29.3 | 26.4 | 60.5 |
| 3 | 15.1 | 13.6 | 74.1 |
| 4 | 14 | 12.7 | 86.8 |
| 5 | 6.01 | 5.4 | 92.2 |
