# Supplementary material for: Fish nursery value of algae habitats in temperate coastal reefs
Source: PeerJ. 2019 May 15;7:e6797. doi: 10.7717/peerj.6797 (PMC6525592; doi:10.7717/peerj.6797)
Supplement: Table S2 — Average squared distance between transects belonging to tested seasons and Islands sampling events. Shaded boxes show average squared distance between transects within season and island grouping. The lager the average squared distance the more dissimilar are the dive transect groupings with respect to their algae cover composition. Bold numbers are representing the highest values measured indicating that transects sampled in summer in Menorca were distinct from the other sampling seasons. [file peerj-07-6797-s009.docx]

| **Island/Season** | Mallorca spring | Mallorca summer | Menorca spring | Menorca Summer |
| --- | --- | --- | --- | --- |
| Mallorca spring | 66.1 |  |  |  |
| Mallorca summer | 180.8 | 68.7 |  |  |
| Menorca spring | 202.2 | 210.6 | 96.1 |  |
| Menorca Summer | **308.2** | **268.8** | **287.1** | 84.3 |
