# Supplementary material for: Fish nursery value of algae habitats in temperate coastal reefs
Source: PeerJ. 2019 May 15;7:e6797. doi: 10.7717/peerj.6797 (PMC6525592; doi:10.7717/peerj.6797)
Supplement: Table S3 — Results of the distance based linear model DistLM relating environmental variables to the PCA ordination shown in Fig. 3. [file peerj-07-6797-s010.docx]

| **Variables** | **SS(trace)** | **Pseudo-F** | **P** |
| --- | --- | --- | --- |
| **TºC** | 1914.1 | 18.856 | 0.001 |
| **Mean depth** | 193.44 | 1.7496 | 0.118 |
| **Wave stress** | 1772.1 | 17.33 | 0.001 |
| **Rugosity** | 479.41 | 4.3958 | 0.002 |
| **Slope** | 96.59 | 0.8696 | 0.468 |
| **Herbivores** | 183.6 | 1.6598 | 0.128 |
| res.df: 190 |  |  |  |
