# Supplementary material for: Fish nursery value of algae habitats in temperate coastal reefs
Source: PeerJ. 2019 May 15;7:e6797. doi: 10.7717/peerj.6797 (PMC6525592; doi:10.7717/peerj.6797)
Supplement: Table S4 — Results of two-way variance analysis on the size of morphotypes and the effect of season tested for both Islands Mallorca and Menorca. Test were run on log transformed height in cm [file peerj-07-6797-s011.docx]

.

| Algae morphotype height comparisons (log transformed) | | | | |  |  |
| --- | --- | --- | --- | --- | --- | --- |
| **Mallorca** | **Df** | **Sum Sq** | **Mean Sq** | **F value** | **Pr(>F)** |  |
| Morf | 5 | 169.83 | 33.97 | 414.7 | <2e-16 |  |
| Per | 1 | 13.72 | 13.72 | 167.5 | <2e-16 | *** |
| Morf:Per | 5 | 49.36 | 9.87 | 120.5 | <2e-16 | *** |
| Residuals | 1085 | 88.87 | 0.08 |  |  | *** |
|  |  |  |  |  |  |  |
| **Menorca** | **Df** | **Sum Sq** | **Mean Sq** | **F value** | **Pr(>F)** |  |
| Morf | 5 | 166.79 | 33.36 | 329.3 | < 2e-16 |  |
| Per | 1 | 3.66 | 3.66 | 36.14 | 2.66E-09 | *** |
| Morf:Per | 5 | 27.97 | 5.59 | 55.21 | < 2e-16 | *** |
| Residuals | 907 | 91.88 | 0.1 |  |  | *** |
