# Supplementary material for: Fish nursery value of algae habitats in temperate coastal reefs
Source: PeerJ. 2019 May 15;7:e6797. doi: 10.7717/peerj.6797 (PMC6525592; doi:10.7717/peerj.6797)
Supplement: Table S5 — Results of the post-hoc Tukey test related to the analyses performed in table S11. Differences in the same morphotypes between seasons in the tow islands and differences between morphotypes within seasons. [file peerj-07-6797-s012.docx]

| **TukeyHSD** | | **Log(Algae height in cm)** | | |  |
| --- | --- | --- | --- | --- | --- |
|  | **Comparisons** | **Mallorca** |  | **Menorca** |  |
| Season | 1-ET | **0.0000** | ******* | **0.0000** | ******* |
| (Spring vs summer) | 2-SL | **0.0000** | ******* | 0.9992 |  |
|  | 3-FI | 0.0552 |  | 0.8632 |  |
|  | 6-BT | 1.0000 |  | 1.0000 |  |
|  | 7-LB | **0.0000** | ******* | **0.0000** | ******* |
|  | 8-TF | **0.0000** | ******* | **0.0001** | ******* |
|  |  |  |  |  |  |
| Spring | 2-SL vs 1-ET | 0.2662 |  | 1.0000 |  |
| (differences between morphotypes) | 3-FI vs 1-ET | **0.0000** | ******* | 0.5779 |  |
|  | 6-BT vs 1-ET | **0.0000** | ******* | 1.0000 |  |
|  | 7-LB vs 1-ET | **0.0000** | ******* | **0.0000** | ******* |
|  | 8-TF vs 1-ET | **0.0000** | ******* | **0.0000** | ******* |
|  | 3-FI vs 2-SL | 0.0067 |  | 0.9815 |  |
|  | 6-BT vs 2-SL | **0.0007** |  | 1.0000 |  |
|  | 7-LB vs 2-SL | **0.0000** | ******* | **0.0000** | ******* |
|  | 8-TF vs 2-SL | **0.0000** | ******* | **0.0000** | ******* |
|  | 6-BT vs 3-FI | 0.9976 |  | 0.6650 |  |
|  | 7-LB vs 3-FI | **0.0000** | ******* | **0.0000** | ******* |
|  | 8-TF vs 3-FI | **0.0000** | ******* | **0.0000** | ******* |
|  | 7-LB vs 6-BT | **0.0000** | ******* | **0.0000** | ******* |
|  | 8-TF vs 6-BT | **0.0000** | ******* | **0.0000** | ******* |
|  | 8-TF vs 7-LB | 1.0000 |  | **0.0000** | ******* |
|  |  |  |  |  |  |
|  | 2-SL vs 1-ET | **0.0000** | ******* | 0.9997 |  |
|  | 3-FI vs 1-ET | **0.0000** | ******* | **0.0000** | ******* |
|  | 6-BT vs 1-ET | **0.0000** | ******* | **0.0000** | ******* |
|  | 7-LB vs 1-ET | **0.0000** | ******* | **0.0000** | ******* |
|  | 8-TF vs 1-ET | **0.0000** | ******* | **0.0000** | ******* |
|  | 3-FI vs 2-SL | **0.0000** | ******* | 0.9990 |  |
|  | 6-BT vs 2-SL | 0.9753 |  | 0.9989 |  |
|  | 7-LB vs 2-SL | 0.1823 |  | 1.0000 |  |
|  | 8-TF vs 2-SL | **0.0000** | ******* | **0.0444** | ***** |
|  | 6-BT vs 3-FI | 0.4220 |  | 1.0000 |  |
|  | 7-LB vs 3-FI | **0.0000** | ******* | 0.9360 |  |
|  | 8-TF vs 3-FI | **0.0000** | ******* | **0.0000** | ******* |
|  | 7-LB vs 6-BT | 0.1331 |  | 0.5911 |  |
|  | 8-TF vs 6-BT | **0.0000** | ******* | **0.0000** | ******* |
|  | 8-TF vs 7-LB | **0.0000** | ******* | **0.0000** | ******* |
