# Supplementary material for: Fish nursery value of algae habitats in temperate coastal reefs
Source: PeerJ. 2019 May 15;7:e6797. doi: 10.7717/peerj.6797 (PMC6525592; doi:10.7717/peerj.6797)
Supplement: Table S6 — Mean abundance (0.062 m 2) and percentage contribution of dominant taxa inhabiting different morphotypes. [file peerj-07-6797-s013.docx]

| Morphotypes | 1-ET | | 2-SL | | 3-FI | | 6-BT | | 7-LB | | 8-TF | |
| --- | --- | --- | --- | --- | --- | --- | --- | --- | --- | --- | --- | --- |
| Taxa: | mean | % | mean | % | mean | % | mean | % | mean | % | mean | % |
| ***Harpacticoida*** | 3146 | **41.4** | 1971 | **39.6** | 1800 | **36.9** | 5066 | **33.9** | 1878 | **35.7** | 2285 | **32.4** |
| ***Gastropoda*** | 447 | 5.9 | 518 | **10.4** | 384 | **7.9** | 2365 | **15.8** | 132 | 2.5 | 447 | 6.3 |
| ***Ostracoda*** | 610 | **8.0** | 660 | **13.3** | 360 | **7.4** | 1100 | **7.4** | 755 | **14.3** | 651 | **9.2** |
| ***Amphipoda*** | 985 | **12.9** | 280 | **5.6** | 369 | **7.6** | 1260 | **8.4** | 217 | 4.1 | 777 | **11.0** |
| ***Bivalvia*** | 542 | **7.1** | 343 | **6.9** | 291 | 6.0 | 1470 | **9.8** | 117 | 2.2 | 371 | 5.3 |
| ***Polychaeta*** | 508 | **6.7** | 187 | 3.8 | 319 | 6.5 | 800 | 5.3 | 270 | 5.1 | 489 | **6.9** |
| ***Acari*** | 186 | 2.4 | 234 | 4.7 | 170 | 3.5 | 657 | 4.4 | 461 | **8.8** | 350 | 5.0 |
| ***Nematoda*** | 81 | 1.1 | 101 | 2.0 | 322 | **6.6** | 503 | 3.4 | 490 | **9.3** | 424 | **6.0** |
| ***Caprellidae*** | 209 | 2.7 | 115 | 2.3 | 259 | 5.3 | 503 | 3.4 | 163 | 3.1 | 152 | 2.2 |
| ***Diptera*** | 109 | 1.4 | 37 | 0.7 | 59 | 1.2 | 311 | 2.1 | 23 | 0.4 | 316 | 4.5 |
| ***Cumacea*** | 83 | 1.1 |  |  | 45 | 0.9 | 47 | 0.3 | 348 | **6.6** | 159 | 2.2 |
| *Tanaidacea* | 138 | 1.8 | 77 | 1.5 | 56 | 1.2 | 194 | 1.3 | 79 | 1.5 | 180 | 2.6 |
| *Isopoda* | 182 | 2.4 | 66 | 1.3 | 80 | 1.6 | 155 | 1.0 | 32 | 0.6 | 89 | 1.3 |
| *Amphiura* | 50 | 0.7 | 50 | 1.0 | 54 | 1.1 | 152 | 1.0 | 21 | 0.4 | 123 | 1.7 |
| *Nudibranchia* | 32 | 0.4 | 12 | 0.2 | 69 | 1.4 | 168 | 1.1 | 21 | 0.4 |  |  |
| *Opisthobranchia* | 2 | 0.0 | 43 | 0.9 | 50 | 1.0 |  |  | 133 | 2.5 | 32 | 0.5 |
| *Pantopoda* | 52 | 0.7 | 17 | 0.3 | 43 | 0.9 | 111 | 0.7 | 18 | 0.3 | 43 | 0.6 |
| *Galathea* | 54 | 0.7 | 25 | 0.5 | 44 | 0.9 | 24 | 0.2 | 30 | 0.6 | 47 | 0.7 |
| *Asteroidea* | 37 | 0.5 | 108 | 2.2 | 29 | 0.6 | 12 | 0.1 | 16 | 0.3 | 13 | 0.2 |
| Others | 150 | 2.0 | 132 | 2.7 | 74 | 1.5 | 65 | 0.4 | 60 | 1.1 | 109 | 1.5 |
| Total | 7604 | 100 | 4976 | 100 | 4876 | 100 | 14963 | 100 | 5266 | 100 | 7056 | 100 |
