# Supplementary material for: Fish nursery value of algae habitats in temperate coastal reefs
Source: PeerJ. 2019 May 15;7:e6797. doi: 10.7717/peerj.6797 (PMC6525592; doi:10.7717/peerj.6797)
Supplement: Table S7 — Results of the one way ANOVA comparing prey availabiltiy within different algae morphotypes [file peerj-07-6797-s014.docx]

.

| ***Diplodus vulgaris*** |  |  |  |  |  |  |
| --- | --- | --- | --- | --- | --- | --- |
| **Abundance** | Df | Sum Sq | Mean Sq | F value | Pr(>F) |  |
| Morphotype | 5 | 30.81 | 6.162 | 4.666 | 0.0006 | *** |
| Residuals | 118 | 155.85 | 1.32 |  |  |  |
|  |  |  |  |  |  |  |
| **Biomass** | Df | Sum Sq | Mean Sq | F value | Pr(>F) |  |
| Morphotype | 5 | 27.52 | 5.505 | 6.398 | 3E-05 | *** |
| Residuals | 118 | 101.52 | 0.86 |  |  |  |
|  |  |  |  |  |  |  |
| ***Coris julis*** |  |  |  |  |  |  |
| **Abundance** | Df | Sum Sq | Mean Sq | F value | Pr(>F) |  |
| Morphotype | 5 | 27.49 | 5.499 | 6.287 | 4E-05 | *** |
| Residuals | 108 | 94.46 | 0.875 |  |  |  |
|  |  |  |  |  |  |  |
| **Biomass** | Df | Sum Sq | Mean Sq | F value | Pr(>F) |  |
| Morphotype | 5 | 35.23 | 7.046 | 8.627 | 7E-07 | *** |
| Residuals | 108 | 88.2 | 0.817 |  |  |  |
|  |  |  |  |  |  |  |
| ***Symphodus occelatus*** |  |  |  |  |  |  |
| **Abundance** | Df | Sum Sq | Mean Sq | F value | Pr(>F) |  |
| Morphotype | 5 | 29.53 | 5.906 | 5.801 | ####### | *** |
| Residuals | 108 | 109.95 | 1.018 |  |  |  |
|  |  |  |  |  |  |  |
| **Biomass** | Df | Sum Sq | Mean Sq | F value | Pr(>F) |  |
| Morphotype | 5 | 38.7 | 7.74 | 9.432 | ####### | *** |
