# Supplementary material for: Fish nursery value of algae habitats in temperate coastal reefs
Source: PeerJ. 2019 May 15;7:e6797. doi: 10.7717/peerj.6797 (PMC6525592; doi:10.7717/peerj.6797)
Supplement: Table S8 — Results of the pairwise comparisons between prey availability (abundance and biomass) in different algae morphotypes for the three study fish species from the post-hoc turkey test. For the original one-way ANOVA analysis results see S14. [file peerj-07-6797-s015.docx]

| ***Diplodus vulgaris*** |  |  |  |  |  |  |  |  |  |  |  |  |
| --- | --- | --- | --- | --- | --- | --- | --- | --- | --- | --- | --- | --- |
| **Abundance** | **1-ET** | **2-SL** | **3-FI** | **6-BT** | **7-LB** |  | **Biomass** | **1-ET** | **2-SL** | **3-FI** | **6-BT** | **7-LB** |
| **1-ET** |  |  |  |  |  |  | **1-ET** |  |  |  |  |  |
| **2-SL** | 0.3984 |  |  |  |  |  | **2-SL** | 0.2547 |  |  |  |  |
| **3-FI** | 0.2233 | 1.0000 |  |  |  |  | **3-FI** | 0.2068 | 1.0000 |  |  |  |
| **6-BT** | 0.9654 | 0.1023 | **0.0360** |  |  |  | **6-BT** | 0.2819 | **0.0012** | **0.0004** |  |  |
| **7-LB** | **0.0083** | 0.5207 | 0.4871 | **0.0011** |  |  | **7-LB** | 0.0745 | 0.9682 | 0.9246 | **0.0004** |  |
| **8-TF** | 0.7563 | 0.9876 | 0.9646 | 0.2909 | 0.1774 |  | **8-TF** | 0.9552 | 0.7596 | 0.7589 | **0.0494** | 0.3449 |
|  |  |  |  |  |  |  |  |  |  |  |  |  |
| ***Coris julis*** |  |  |  |  |  |  |  |  |  |  |  |  |
| **Abundance** | **1-ET** | **2-SL** | **3-FI** | **6-BT** | **7-LB** |  | **Biomass** | **1-ET** | **2-SL** | **3-FI** | **6-BT** | **7-LB** |
| **1-ET** |  |  |  |  |  |  | **1-ET** |  |  |  |  |  |
| **2-SL** | 0.1913 |  |  |  |  |  | **2-SL** | **0.0109** |  |  |  |  |
| **3-FI** | 0.7279 | 0.9437 |  |  |  |  | **3-FI** | **0.0289** | 0.9991 |  |  |  |
| **6-BT** | 0.2458 | **0.0005** | **0.0105** |  |  |  | **6-BT** | 0.3902 | **0.0000** | **0.0001** |  |  |
| **7-LB** | 0.7321 | 0.7983 | 0.9999 | **0.0036** |  |  | **7-LB** | 0.3484 | 0.3988 | 0.6455 | **0.0013** |  |
| **8-TF** | 0.8978 | 0.7069 | 0.9975 | **0.0161** | 0.9998 |  | **8-TF** | 0.9742 | 0.0591 | 0.1358 | 0.0780 | 0.8125 |
|  |  |  |  |  |  |  |  |  |  |  |  |  |
| ***Symphodus occelatus*** |  |  |  |  |  |  |  |  |  |  |  |  |
| **Abundance** | **1-ET** | **2-SL** | **3-FI** | **6-BT** | **7-LB** |  | **Biomass** | **1-ET** | **2-SL** | **3-FI** | **6-BT** | **7-LB** |
| **1-ET** |  |  |  |  |  |  | **1-ET** |  |  |  |  |  |
| **2-SL** | 0.1421 |  |  |  |  |  | **2-SL** | **0.0153** |  |  |  |  |
| **3-FI** | 0.7937 | 0.8536 |  |  |  |  | **3-FI** | 0.4747 | 0.6685 |  |  |  |
| **6-BT** | 0.1315 | **0.0001** | **0.0052** |  |  |  | **6-BT** | 0.1543 | **0.0000** | **0.0011** |  |  |
| **7-LB** | 0.7562 | 0.6799 | 1.0000 | **0.0010** |  |  | **7-LB** | 0.1322 | 0.7586 | 0.9990 | **0.0000** |  |
| **8-TF** | 0.9999 | 0.0764 | 0.6477 | 0.1845 | 0.5690 |  | **8-TF** | 0.9768 | **0.0012** | 0.1228 | 0.4908 | **0.0113** |
