# Supplementary figures and images for: Fish nursery value of algae habitats in temperate coastal reefs
Source: PeerJ. 2019 May 15;7:e6797. doi: 10.7717/peerj.6797 (PMC6525592; doi:10.7717/peerj.6797)

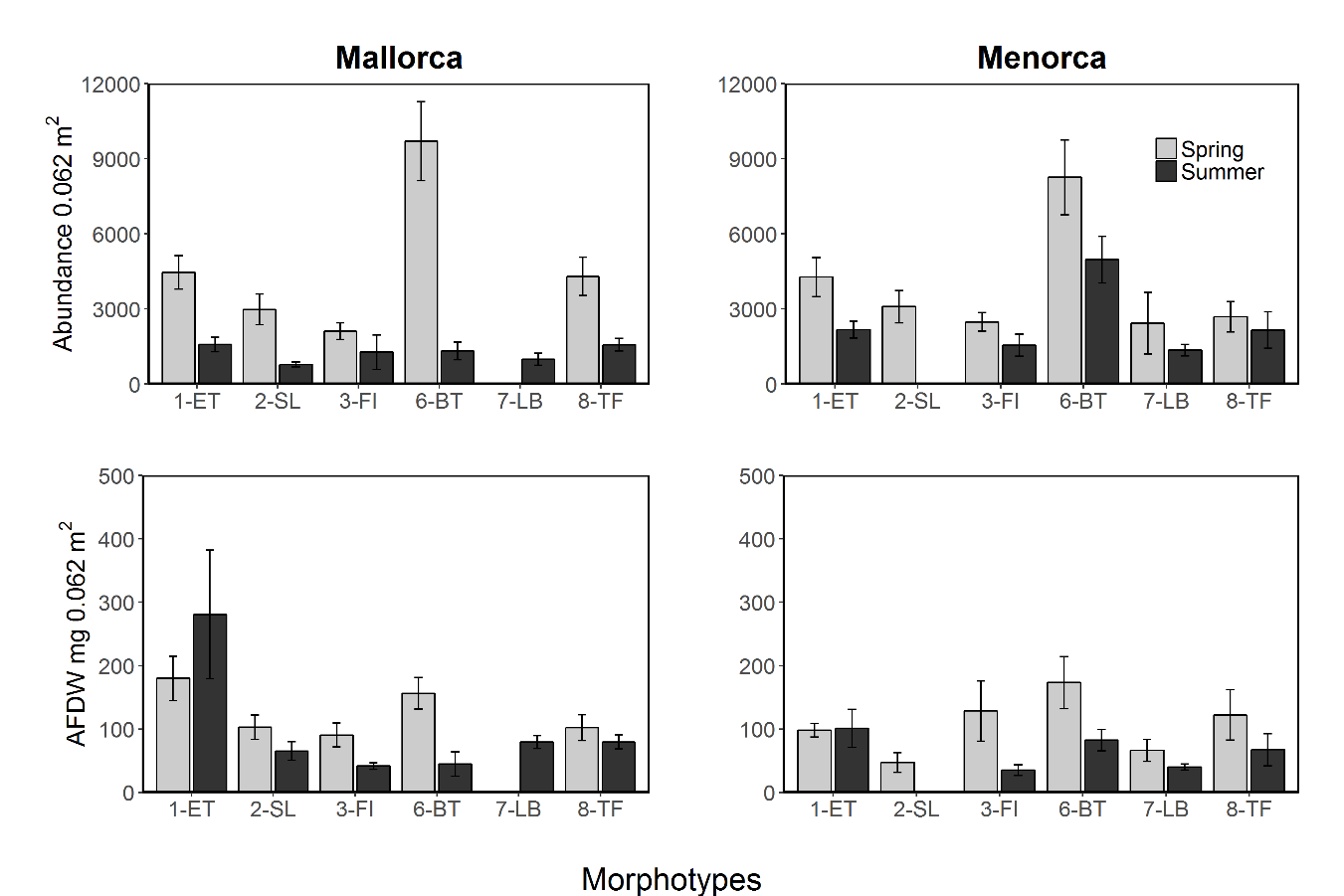

Supplement: Figure S1 — Abundance (0.062 m2) and estimated biomass (AFDW mg per 0.062 m2) of invertebrate fauna associated to different algae morphotypes collected in spring (May 2014) and summer (August 2014) in Mallorca and Menorca. Error bar are S.E. [file peerj-07-6797-s016.png]
